# Methamphetamine-related psychiatric emergencies in Türkiye: clinical features, risk factors, and follow-up results from a tertiary mental health center

**DOI:** 10.3389/fpsyt.2025.1675959

**Published:** 2025-10-09

**Authors:** Mine Ergelen Yalçın, Salih Cihat Paltun

**Affiliations:** Adult Alcohol and Substance Use Disorders Detoxification Center, Erenköy Mental and Nervous Diseases Training and Research Hospital, University of Health Sciences, Istanbul, Türkiye

**Keywords:** methamphetamine, suicide, psychosis, comorbidity, emergency services, psychiatric, Türkiye

## Abstract

**Objectives:**

Methamphetamine (MA) use is a growing public health issue in Türkiye, leading to an increasing number of psychiatric emergencies. There is limited information on the clinical features and outcomes of MA users in non-Western countries.

**Methods:**

This retrospective cohort study included 423 patients with confirmed MA use among 12,501 psychiatric emergency department (PED) admissions at a tertiary mental health center in Istanbul, Türkiye, between January and June 2022. Data on demographics, clinical presentation, comorbidities, and follow-up outcomes were collected from electronic medical records.

**Results:**

Most patients were young adult males (84.2%). Psychotic symptoms (65.5%), agitation (65.7%), and insomnia (60.8%) were common. Depression (OR = 18.0, 95% CI: 3.7–88.1) and self-harm (OR = 26.5, 95% CI: 7.1–98.9) were the strongest predictors of suicide attempts. Psychotic symptoms (OR = 2.6, 95% CI: 1.5–4.7), agitation (OR = 2.2, 95% CI: 1.3–3.7), and self-harm (OR = 3.5, 95% CI: 1.9–6.6) were linked to aggression. Prior psychiatric hospitalization (OR = 7.4, 95% CI: 4.4–12.3) and comorbid psychiatric disorders (OR = 2.2, 95% CI: 1.3–3.6) predicted frequent PED visits. Within one year, 33.3% of patients were hospitalized.

**Conclusions:**

MA users admitted to psychiatric emergency services in Türkiye often present with severe symptoms, high rates of comorbidity, and polysubstance use. Recognizing key risk factors may help guide early intervention and integrated care for this vulnerable group. These findings add new knowledge from a non-Western context and may inform clinical practice and policy in similar settings worldwide.

## 1 Introduction

The illicit use of methamphetamine (MA)—a powerful stimulant often referred to as ‘crystal meth’, ‘ice’, or ‘speed’—has become a significant public health concern worldwide (1). Its pharmacological properties, including rapid onset, long half-life, and strong central nervous system stimulation, have contributed to its widespread misuse and the complex clinical challenges (2). As a result, MA use creates significant burdens for psychiatric emergency departments (PEDs) due to its psychiatric and behavioral consequences. Rising rates of MA use have led to increased PED presentations globally. This global surge is particularly evident in Türkiye, which accounted for a significant proportion of methamphetamine treatment admissions in Europe in 2021. In 2022, amphetamines or methamphetamines similarly accounted for at least 15% of first-time treatment entrants in Türkiye and neighbouring or regional countries such as Bulgaria and several Central and Eastern European states (3). These figures underline Türkiye’s position as one of the key countries affected by MA use in the wider region, making it essential to examine its local impact in detail.

The COVID-19 pandemic has further strained psychiatric care and addiction treatment systems, intensifying the burden of stimulant-related emergencies (4). In countries such as Türkiye, the availability and increased potency of MA have contributed to growing numbers of individuals presenting with acute psychiatric disturbances (5, 6).

MA use produces a wide spectrum of psychiatric and behavioral effects. Adverse outcomes include dysphoria, anxiety, insomnia, depression, and suicidal ideation. Studies have documented that a significant proportion of individuals develop psychotic symptoms such as delusions, paranoia, and hallucinations (7, 8). In addition, hostility, deliberate self-harm, and aggressive or violent behavior are frequently reported in this population (9, 10). While these manifestations are well documented, their severity and pattern vary considerably across patients. Factors such as comorbid psychiatric disorders and concurrent use of other substances further influence the clinical presentation (11, 12).

Importantly, these psychiatric consequences of MA use have also been documented in non-Western contexts. In Iran, MA use has become one of the most pressing substance related public health challenges, with persistently high rates of MA induced psychosis reported in treatment settings (7). Data from China indicate that more than half of individuals with MA use present with at least one psychiatric symptom — most commonly psychosis, depression, or anxiety — highlighting the substantial clinical burden (13). Reports from Saudi Arabia also describe high levels of suicidality and aggression among MA users (10, 14), further underscoring the severity of presentations in Middle Eastern populations.

The rising prevalence of MA use in diverse sociocultural contexts has placed increasing pressure on psychiatric emergency departments (PEDs). PEDs often serve as the first point of contact for individuals experiencing acute psychiatric symptoms—including agitation, psychosis, and severe mood disturbances—related to MA (15) These emergencies are frequently accompanied by behavioral dysregulation and psychotic features that endanger patient safety and complicate acute management. Consequently, MA-related presentations create substantial challenges for mental health professionals, driving increased use of prehospital, emergency, and inpatient services, and contributing to recurrent episodes of violence directed at staff (16–19).

Literature shows a marked escalation in MA-related admissions to treatment facilities and emergency departments over the past decade (20, 21). Compared to non-users, MA users exhibit increased frequency of visits, longer hospital stays, elevated in-hospital mortality rates, more involuntary psychiatric holds, greater use of physical restraints, and overall greater healthcare resources (21–23). These patterns are largely driven by comorbidities such as psychotic disorders, substance-related psychosis, concurrent use of other illicit drugs, suicidal ideation or attempts, aggression, and deliberate self-harm, all of which worsen clinical outcomes and increase service needs (15, 19, 24–28). Understanding these factors is essential to improving care quality in psychiatric emergency settings (1), including reducing violence, promoting safety, minimizing the use of restrictive measures like chemical and physical restraints and supporting more effective therapeutic relationships (29). Such efforts may ultimately enhance adherence and outcomes in conditions like psychotic and substance-induced psychotic disorders, as well as suicidality. Beyond acute psychiatric effects, MA use is linked to significant social consequences, including stigma, reduced access to non-acute healthcare due to confidentiality concerns, housing instability, and criminal justice involvement, exacerbating social and health disparities (1).

This study was conducted at a tertiary mental health center in Istanbul, a major referral institution for psychiatric emergencies in the region. Given Türkiye’s unique position bridging Europe and Middle East, examining the clinical characteristics and outcomes of MA users in this context can offer valuable insights for both regional and international mental health strategies for PEDs, especially in countries facing similar public health challenges.

Specific data on the clinical presentation and risk factors among methamphetamine (MA) users remain limited in non-Western regions, particularly in Türkiye. As the country undergoes rapid sociocultural change and rising substance abuse, it faces significant mental health challenges and thus warrants special attention (30). At the same time, emerging evidence from neighboring non-Western settings—including Iran, China, and Saudi Arabia—already documents severe psychiatric and social consequences of MA use (7, 10, 13, 14). Türkiye’s strategic location, influenced by both Middle Eastern and European cultures, shapes unique societal norms and contributes to the stigma surrounding mental health and substance use. Identifying the clinical patterns and risk factors associated with MA use in this setting is essential for improving patient care, informing policy, and allocating healthcare resources effectively.

Accordingly, the present study investigates the demographic characteristics, acute psychiatric manifestations, comorbidities, and clinical outcomes of MA users admitted to a psychiatric emergency department (PED) in Türkiye. We hypothesized that (i) psychotic symptoms would be predominant in the majority of patients presenting to a tertiary PED in Türkiye due to MA-related reasons, and that (ii) age, gender, polydrug use, and the presence of psychotic symptoms at admission would be related to short- and medium-term adverse outcomes of MA-related emergency visits, including suicidal ideation, suicide attempt, aggression, repeated PED visits, and one-year psychiatric hospitalization. Our objective was to delineate the clinical features and risk factors of MA-related psychiatric emergencies in Türkiye in order to inform tailored interventions and strengthen emergency mental health services.

## 2 Methods

This retrospective cohort study was conducted at the PED of Erenköy Mental and Nervous Diseases Training and Research Hospital (ERSHEAH) in Istanbul, Türkiye. ERSHEAH is a major mental health center serving a catchment area of approximately 6.3 million people and includes a dedicated Alcohol and Substance Use Disorders Detoxification Centre (ASUDDC). The PED receives around 25,000 visits annually from individuals who present voluntarily, via emergency medical services, or through compulsory referrals.

Electronic medical records from January 1 to June 30, 2022, were reviewed. During this period, 12,501 patient records were screened, and 423 individuals aged 18 years or older who presented with MA use were included in the study. MA use was established on the basis of a documented positive urine toxicology screen when available, or otherwise on a clear patient self-report of MA use recorded in the medical file. No additional exclusion criteria were applied. The study period (January-June 2022) was selected as it represented a post-COVID-19 pandemic interval, minimizing the influence of major public health restrictions and other significant external events on psychiatric emergency admissions.

Data were extracted between September and October 2024. For each patient, sociodemographic features, mode of arrival, presenting symptoms, concurrent substance use, comorbidities, criminal history, and clinical features at admission and after one year were documented. The frequency of emergency visits, suicidal ideation or behavior, and aggression at admission were also recorded. The assignment of symptoms was based on the explicit clinical findings recorded by the attending clinicians in the hospital charts during routine psychiatric examinations. Follow-up variables were extracted from the same electronic medical registry and included subsequent PED visits, hospitalizations, and diagnostic updates recorded within one year after the index admission of each patient, but symptom-level detail was not consistently available at follow-up. The variable ‘three or more PED visits’ was coded to include the index admission as well as any subsequent PED presentations during the one-year follow-up period.

Diagnoses were classified according to the International Classification of Diseases, 10th Revision (ICD-10). Psychotic disorders were coded as F20–F29, depressive disorders as F32 and F33, and anxiety disorders as F40 and F41. Bipolar disorder diagnoses included all relevant mood episodes and remission states.

All statistical analyses were performed using SPSS (IBM Corp., Armonk, NY, USA). Continuous variables are presented as means and standard deviations, while categorical variables are reported as percentages. Associations between categorical variables were assessed using Pearson’s Chi-square test. Logistic regression analyses were conducted to identify factors associated with suicidal ideation and attempts, self-harm, aggressive behavior, frequent PED visits, psychiatric hospitalization within one year of PED admission, and substance-induced psychosis at one-year follow-up. The selection of predictor variables for each logistic regression model was guided by both clinical relevance and univariate associations, reflecting the distinct conceptual and empirical underpinnings of outcomes such as suicidality versus non-suicidal self-harm. Results are reported as odds ratios (OR) with 95% confidence intervals (CI). Statistical significance was set at p < 0.05.

## 3 Results

### 3.1 Sociodemographic and admission characteristics

A total of 423 patients were included in the study, with 84.2% (n=356) being male and 15.8% (n=67) female. The mean age was 30.1 years (SD = 7.2). Among the patients, 27.9% (n=118) arrived by ambulance, and 19.1% (n=81) were brought in by police referral. At admission, 80.6% (n=341) of the patients were accompanied by a relative, while 19.4% (n=82) presented alone.

### 3.2 Clinical presentation at PED admission

Psychotic symptoms were present in 65.5% (n=277) of the patients. Visual hallucinations were observed in 19.1% (n=81), auditory hallucinations in 38.8% (n=164), and tactile hallucinations in 0.2% (n=1). Persecutory delusions were detected in 54.8% (n=232), delusions of reference in 31.7% (n=134), grandiose delusions in 6.4% (n=27), somatic delusions in 1.7% (n=7), and jealous delusions in 6.6% (n=28). Dissociative symptoms were present in 21.7% (n=92), with amnesia in 1.2% (n=5), depersonalization in 16.1% (n=68), and derealization in 4.5% (n=19). Suicidal ideation was present in 9.9% (n=42) and suicide attempt in 5% (n=21) of the patients. Aggression was observed in 34.3% (n=145), including personal aggression (28.4%, n=120), property damage (27.7%, n=117), and self-harm (14.4%, n=61). Insomnia was seen in 60.8% (n=257) and agitation in 65.7% (n=278).

### 3.3 Use patterns and psychiatric comorbidities

Among MA users, 16.5% used alcohol, 38.5% used cannabis, 29.8% used synthetic cannabinoids, 12.3% used heroin, and 6.6% used cocaine. Additionally, 9.7% of the patients were using three or more psychoactive substances. Of the patients, 55.8% (n=236) had a psychiatric disorder comorbidity. Specifically, 1.7% (n=7) had schizophrenia, 8% (n=34) had bipolar disorder, 9.2% (n=39) had depression, 2.6% (n=11) were diagnosed with an anxiety disorder, 15.4% (n=65) had other psychotic disorders, 9% (n=38) had substance-related psychosis, and 9.9% (n=42) had other psychiatric disorders. These comorbidities represent previously established diagnoses documented in the medical records prior to the index admission and confirmed during the emergency evaluation. **Table 1** presents the clinical characteristics of the patients at PED admission.

**Table 1:** Clinical characteristics of the patients at the PED admission (n=423).

Variable	Group	n	%
Gender	Woman Man	67 356	15.8 84.2
Admission	Alone with Relatives	82 341	19.4 80.6
Ambulance referral	Yes No	118 305	27.9 72.1
Law enforcement referral	Yes No	81 342	19.1 80.9
Symptoms at the PED admission			
One or more psychotic symptom	Yes No	277 146	65.5 34.5
Auditory hallucination	Yes No	164 259	38.8 61.2
Visual hallucination	Yes No	81 342	19.1 80.9
Tactile hallucination	Yes No	1 422	0.2 99.8
Persecutory delusion	Yes No	232 191	54.8 45.2
Delusion of reference	Yes No	134 289	31.7 68.3
Grandiose delusion	Yes No	27 396	6.4 93.6
Somatic delusion	Yes No	7 416	1.7 98.3
Delusion of jealousy	Yes No	28 395	6.6 93.4
Dissociative symptom	No Amnesia Depersonalization Derealization	331 5 68 19	78.3 1.2 16.1 4.5
Suicidal ideation	Yes No	42 381	9.9 90.1
Suicide attempt	Yes No	21 402	5.0 95.0
Aggression	Yes No	145 278	34.3 65.7
Self-Harm	Yes No	61 362	14.4 85.6
Insomnia	Yes No	257 166	60.8 39.2
Agitation	Yes No	278 145	65.7 34.3
Concurrent alcohol and drug use			
Concurrent drug use with MA	Yes No	324 99	76.6 23.4
Use of three or more drug	Yes No	41 382	9.7 90.3
Alcohol use	Yes No	70 353	16.5 83.5
Cannabis use	Yes No	163 260	38.5 61.5
Synthetic cannabinoid use	Yes No	126 297	29.8 70.2
Heroin use	Yes No	52 371	12.3 87.7
Cocaine use	Yes No	28 395	6.6 93.4
Use of other drugs	Yes No	50 373	11.8 88.2
Other clinical characteristics of the patients			
Comorbidity of psychiatric disorder	No Schizophrenia Bipolar disorder Depression Anxiety disorder Substance-induced psychosis Psychotic disorder not otherwise specified Other (personality disorders, obsessive compulsive related disorders, eating disorders etc.)	187 7 34 39 11 38 65 42	44.2 1.7 8.0 9.2 2.6 9.0 15.4 9.9
Chronic medical illness	Yes No	22 401	5.2 94.8
Criminal history	Yes No	127 296	30.0 70.0

PED, Psychiatric Emergency Department; n, Number; MA, Methamphetamine.

### 3.4 Treatment and short-term outcomes

Among the treatments given in the emergency department, 63.1% (n=267) of patients were administered parenteral antipsychotics (APs), 21% (n=89) were given benzodiazepines, 3.1% (n=13) were prescribed oral APs, and 12.8% (n=54) received other pharmacologic treatments. Physical restraint was required for 23.6% (n=100) of patients, and observation was necessary for 71.1% (n=300).

Analysis of the treatment outcomes for patients in the emergency department revealed that 63.5% (n=180) were referred to the outpatient clinic of the Alcohol and Substance Use Disorders Detoxification Centre (ASUDDC) of the hospital, 33.9% (n=143) were mandated for compulsory hospitalization, and 2.6% (n=11) opted for voluntary hospitalization.

### 3.5 One-year follow-up outcomes

During follow-up, 33.3% (n=114) of patients required psychiatric hospitalization within one year. Among those with available follow-up data (n=342), 24.6% (n=84) had no psychiatric diagnosis, 2.6% (n=9) were diagnosed with schizophrenia, 9.9% (n=34) with bipolar disorder, 7.6% (n=26) with depression, 2.0% (n=7) with anxiety disorder, 25.4% (n=87) with other psychotic disorders, 3.5% (n=12) with other psychiatric disorders, and 24.3% (n=83) with substance-induced psychosis. **Table 2** presents the clinical characteristics of patients at PED admission and at one-year follow-up.

**Table 2:** Clinical characteristics at the PED admission (n=423).

Variable	Group	n	%
Pharmacological treatment applied in the PED	Oral AP Parenteral AP Benzodiazepine Other	13 267 89 54	3.1 63.1 21.0 12.8
Physical restraint	Yes No	100 323	23.6 76.4
Observation	Yes No	300 122	71.1 28.9
Treatment outcome	Specialized ASUDDC referral Compulsory hospitalization Voluntary hospitalization	268 143 11	63.5 33.9 2.6
Clinical characteristics at the end of one year follow-up after PED admission (n=342)			
Psychiatric hospitalization within one year of PED admission	Yes No	114 228	33.3 66.6
Psychiatric diagnosis after one year follow-up	No Schizophrenia Bipolar disorder Depression Anxiety disorder Substance-induced psychosis Psychotic disorder not otherwise specified Other (personality disorders, obsessive compulsive related disorders, eating disorders etc.)	84 9 34 26 7 83 87 12	24.6 2.6 9.9 7.6 2.0 24.3 25.4 3.5

PED, Psychiatric Emergency Department; n, Number; AP, Antipsychotic; ASUDDC, Alcohol and Substance Use Disorders Detoxification Center.

### 3.6 Previous treatment history

Upon analyzing the past treatment history, 43.5% (n=184) had visited the emergency department for the first time, 24.8% (n=105) had visited once or twice, and 31.7% (n=134) had visited three or more times. Additionally, 64.3% (n=272) had previously visited the ASUDDC outpatient clinic, and 29.4% (n=124) had received inpatient treatment in a psychiatric service before their current emergency visit.

### 3.7 Predictors of suicidal ideation

Multivariate logistic regression analysis revealed a statistically significant relation between suicidal ideation and the presence of psychotic symptoms (OR = 4.83, 95% CI: 1.65–14.19, p = .004), depression (OR = 15.63, 95% CI: 5.30–46.12, p <.001), and self-mutilation (OR = 5.40, 95% CI: 2.41–12.10, p <.001) among patients with methamphetamine use (**Table 3**).

**Table 3:** Multivariate logistic regression analysis of independent risk factors for suicidal ideation.

Predictors of suicidal ideation	β	S.E.	Wald	p	OR	95% CI
Age	-.003	.025	.012	.911	.997	[.950-1.046]
Gender	-.275	.534	.264	.607	.760	[.267-2.166]
Concurrent drug use with MA	-.082	.438	.035	.852	.922	[.391-2.172]
Use of more than three drugs	.612	.617	.983	.322	. 1.846	[.550-6.173]
One or more psychotic symptom	1.575	.550	8.213	.004*	4.832	[1.645-14.191]
Auditory hallucination	-.542	.400	1.833	.176	.582	[.266-1.274]
Insomnia	-.057	.402	.020	.887	.944	[.430-2.076]
Agitation	-.236	.413	.325	.569	.790	[.352-1.776]
Self-harm	1.685	.412	16.733	.000*	5.395	[2.406-12.097]
Comorbidity of psychiatric disorder	.031	.398	.006	.938	1.032	[.473-2.252]
Depression	2.749	.552	24.800	.000*	15.631	[5.297-46-122]
Substance-induced psychosis	.675	.811	.692	.405	1.963	[.401-9.602]
Three or more PED visits	-.184	.385	.228	.633	1.202	[0.565-2.557]

PED, Psychiatric Emergency Department; MA, Methamphetamine. *p < 0.05; bold values also indicate statistical significance.

### 3.8 Predictors of suicide attempt

For suicide attempts, multivariate analysis showed that depression (OR = 18.01, 95% CI: 3.68–88.09, p <.001), comorbidity of psychiatric disorder (OR = 0.16, 95% CI: 0.03–0.80, p = .026), and self-mutilation (OR = 26.54, 95% CI: 7.12–98.87, p <.001) were significant predictors (**Table 4**).

**Table 4:** Multivariate logistic regression analysis of independent risk factors for suicide attempt.

Predictors of suicide attempt	β	S.E.	Wald	p	OR	95% CI
Age	.053	.041	1.624	.203	1.054	[.972-1.143]
Gender	-.106	.744	.020	.887	.900	[.209-3.867]
Use of concurrent drug with MA	.051	.673	.006	.939	1.053	[.282-3.935]
Use of more than three drugs	1.295	.921	1.979	.159	. 3.650	[.601-22.222]
One or more psychotic symptom	1.480	.959	2.385	.123	4.395	[.671-28.769]
Auditory hallucination	.488	.649	.565	.452	1.629	[.456-5.818]
Insomnia	-.764	.622	1.506	.220	.466	[.138-1.578]
Agitation	-.901	.651	1.919	.166	.406	[.113-1.454]
Self-Harm	3.279	.671	23.866	.000*	26.536	[7.122-98.872]
Comorbidity of psychiatric disorder	1.866	.837	4.970	.026*	6.452	[1.253-33.333]
Depression	2.891	.810	12.735	.000*	18.006	[3.680-88.088]
Substance-induced psychosis	.279	.870	.103	.748	1.322	[.240-7.272]
Three or more PED visits	.433	.567	.582	.445	1.540	[0.508-4.695]

PED, Psychiatric Emergency Department; MA, Methamphetamine. *p < 0.05; bold values also indicate statistical significance.

### 3.9 Predictors of self-harm

The concurrent use of drugs with MA (OR = 2.05, 95% CI: 1.08–3.88, p = .028), the presence of one or more psychotic symptoms (OR = 2.25, 95% CI: 1.13–4.46, p = .020), insomnia (OR = 2.60, 95% CI: 1.30–5.22, p = .007), and agitation (OR = 2.93, 95% CI: 1.38–6.19, p = .005) were found to be significant independent predictors of self-harm (**Table 5**).

**Table 5:** Multivariate logistic regression analysis of independent risk factors for self-harm.

Predictors of self-harm	β	S.E.	Wald	p	OR	95% CI
Age	.036	.021	2.849	.091	1.037	[.994-1.081]
Gender	-.423	.423	,997	.318	.655	[.286-1.502]
Concurrent drug use with MA	.717	.326	4.850	.028*	.2.049	[1.082-3.876]
Use of more than three drugs	.900	.645	1.947	.163	2.458	[.695-8.696]
One or more psychotic symptom	.810	.349	5.381	.020*	2.247	[1.134-4.455]
Comorbidity of psychiatric disorder	.259	.321	.650	.420	1.295	[0.690- 2.432]
Insomnia	.956	.356	7.238	.007*	2.602	[1.297-5.224]
Agitation	1.074	.382	7.892	.005*	2.926	[1.383-6.189]
Substance-induced psychosis	.283	.602	.221	.638	.891	[.408-4.321]
Criminal history	-.630	.358	3.090	.079	.533	[.264-1.075]
Three or more PED visits	-.105	.326	.104	.747	1.490	[0.475- 1.704]

PED, Psychiatric Emergency Department; MA, Methamphetamine. *p < 0.05; bold values also indicate statistical significance.

### 3.10 Predictors of aggression

Multivariate regression analysis identified psychotic symptoms (OR = 2.65, 95% CI: 1.49–4.72, p = .001), agitation (OR = 2.24, 95% CI: 1.34–3.74, p = .002), and self-mutilation (OR = 3.54, 95% CI: 1.89–6.63, p <.001) as significant predictors of aggression (**Table 6**).

**Table 6:** Multivariate logistic regression analysis of independent risk factors for aggression.

Predictors of aggression	β	S.E.	Wald	p	OR	95% CI
Age	.017	.016	1.147	.284	1.018	[.986-1.050]
Gender	-.307	.338	.825	.364	.735	[.379-1.427]
Concurrent drug use with MA	.240	.282	.721	.396	1.271	[.731-2.211]
Use of more than three drugs	-.118	.377	.098	.755	0.889	[0.424- 1.863]
One or more psychotic symptom	.974	.295	10.932	.001*	2.649	[1.487-4.718]
Auditory hallucination	.277	.260	1.139	.286	1.319	[.793-2.195]
Insomnia	.285	.243	1.375	.241	1.329	[.826-2.139]
Agitation	.807	.261	9.567	.002*	2.240	[1.344-3.735]
Self-Harm	1.264	.320	15.579	.000*	3.540	[1.890-6.633]
Substance-induced psychosis	-.115	.391	.087	.768	.891	[.414-1.919]
Criminal history	.472	.249	3.588	.058	1.603	[.984-2.612]
Three or more PED visits	.399	.241	2.739	.098	1.490	[0.929- 2.392]

PED, Psychiatric Emergency Department; MA, Methamphetamine. *p < 0.05; bold values also indicate statistical significance.

### 3.11 Predictors of physical restraint

The presence of psychotic symptoms (OR = 6.10, 95% CI: 2.96–12.59, p <.001), self-harm (OR = 4.47, 95% CI: 2.32–8.63, p <.001), and agitation (OR = 3.21, 95% CI: 1.68–6.15, p <.001) were significant predictors of the need for physical restraint (**Table 7**).

**Table 7:** Multivariate logistic regression analysis of independent risk factors for physical restraint.

Predictors of physical restraint	β	S.E.	Wald	p	OR	95% CI
Age	.005	.018	.065	.798	1.005	[.970-1.041]
Gender	.610	.364	2.815	.093	1.841	[.903-3.754]
Concurrent drug use with MA	.040	.319	.016	.899	.488	[.557-1.946]
Use of more than three drugs	.292	.441	.438	.508	1.399	[.564-3.180]
One or more psychotic symptom	1.808	.370	23.929	.000*	6.099	[2.955-12.586]
Comorbid psychiatric disorder	.069	.288	.057	.811	1.072	[0.609- 1.887]
Self-Harm	1.498	.335	19.984	.000*	4.472	[2.319-8.625]
Insomnia	1.167	.332	12.368	.782	.924	[.529-1.615]
Agitation	1.167	.332	12.368	.000*	3.212	[1.676-6.154]
Substance-induced psychosis	-.306	.450	.461	.497	.737	[.305-1.779]
Criminal history	.464	.286	2.634	.105	1.590	[.908-2.784]
Three or more PED visits	.465	.278	2.797	.094	1.594	[0.923- 2.747]

PED, Psychiatric Emergency Department; MA, Methamphetamine. *p < 0.05; bold values also indicate statistical significance.

### 3.12 Predictors of frequent PED visits

Comorbidity of psychiatric disorder (OR = 2.15, 95% CI: 1.27–3.63, p = .004) and prior history of in a closed psychiatric ward (OR = 7.35, 95% CI: 4.39–12.31, p <.001) were significant predictors of three or more PED visits (**Table 8**).

**Table 8:** Multivariate logistic regression analysis of independent risk factors for frequent PED visit (three or more PED visits).

Predictors of Three or more PED visits	β	S.E.	Wald	p	OR	95% CI
Age	-.002	.017	.010	.920	0.998	[0.966- 1.033]
Gender	.306	.338	.819	.365	1.359	[0.700- 2.638]
Concurrent drug use with MA	.347	.301	1.330	.249	1.415	[0.784- 2.551]
Use of more than three drugs	.331	.413	.644	.422	1.393	[.620-3.130]
One or more psychotic symptom	.060	.273	.048	.826	.942	[.551-1.609]
Comorbid psychiatric disorder	.766	.267	8.300	.004*	2.150	[1.274-3.628]
Self-Harm	.003	.358	.000	.994	1.003	[0.497- 2.024]
Insomnia	-.043	.263	.027	.871	0.958	[0.573- 1.603]
Agitation	-.213	.272	.613	.434	0.808	[0.475- 1.377]
Aggression	.341	.272	1.574	.210	1.406	[0.825- 2.392]
Drug-induced psychosis	.113	.416	.073	.787	1.120	[0.495- 2.525]
Criminal history	-.022	.274	.006	.937	0.978	[0.571- 1.675]
Prior history of hospitalization in a closed psychiatric ward	1.995	.263	57.579	.000*	7.350	[4.391-12.305]

PED, Psychiatric Emergency Department; MA, Methamphetamine. *p < 0.05; bold values also indicate statistical significance.

### 3.13 Predictors of psychiatric hospitalization within one year

Older age (OR = 1.04, 95% CI: 1.01–1.08, p = .017) and prior history of hospitalization in a closed psychiatric ward (OR = 4.48, 95% CI: 2.67–7.58, p <.001) were significant predictors of psychiatric hospitalization within one year after PED admission (**Table 9**).

**Table 9:** Multivariate logistic regression analysis of independent risk factors for psychiatric hospitalization in a closed ward within one year of PED admission.

Predictors of psychiatric hospitalization within one year of PED admission	β	S.E.	Wald	p	OR	95% CI
Age	.043	.018	5.663	.017*	1.044	[1.008-1.081]
Gender	.027	.332	.007	.935	1.028	[.536-1.970]
Concurrent drug use with MA	.213	.294	.524	.469	1.237	[.696-2.200]
Use of more than three drug	.076	.424	.032	.857	1.079	[.470-2.476]
One or more psychotic symptom	.175	.272	.415	.519	1.191	[.699-2.030]
Comorbid psychiatric disorder	.348	.264	1.737	.188	1.417	[0.844- 2.381]
Self-Harm	.533	.333	2.551	.110	1.703	[.886-3.275]
Agitation	.024	.265	.008	.068	2.244	[.609-1.722]
Aggression	.434	.262	2.759	.097	.1.544	[.925-2.578]
Substance-induced psychosis	.808	.443	3.333	.068	.2.244	[.942-5.346]
Criminal history	.348	.264	1.738	.187	1.416	[.844-2.377]
Prior history of hospitalization in a closed psychiatric ward	1.502	.266	31.904	.000*	4.484	[2.667- 7.576]

PED, Psychiatric Emergency Department; MA, Methamphetamine. *p < 0.05; bold values also indicate statistical significance.

### 3.14 Predictors of substance-induced psychosis at one-year follow-up

Male gender (OR = 0.23, 95% CI: 0.09–0.63, p = .004), the presence of psychotic symptoms at admission (OR = 2.96, 95% CI: 1.52–5.74, p = .001), agitation (OR = 2.04, 95% CI: 1.12–3.74, p = .020), aggression (OR = 1.84, 95% CI: 1.08–3.15, p = .026), and a prior history of closed ward (OR = 1.92, 95% CI: 1.12–3.30, p = .018) were significant predictors of substance-induced psychosis at one-year follow-up (**Table 10**).

**Table 10:** Multivariate logistic regression analysis of independent risk factors for substance-induced psychosis diagnosis at one year follow-up.

Predictors of substance-induced psychosis diagnosis at follow-up	β	S.E.	Wald	p	OR	95% CI
Age	.035	.019	3.385	.066	1.036	[.998-1.076]
Gender	-1.457	.511	8.132	.004*	.233	[.086-.634]
Concurrent drug use with MA	.312	.335	.865	.352	1.366	[.708-2.633]
Use of more than three drugs	.041	.418	.010	.922	1.042	[.459-2.364]
One or more psychotic symptom	1.084	.339	10.224	.001*	.2.956	[1.521-5.744]
Self-Harm	-.340	.375	.825	.364	.712	[.341-1.483]
Agitation	.715	.308	5.388	.020*	2.043	[1.118-3.735]
Aggression	.612	.274	4.984	.026*	1.843	[1.077-3.153]
Criminal history	.053	.276	.037	.848	1.054	[.614-1.811]
Prior history of hospitalization in a closed psychiatric ward	.653	.276	5.582	.018*	1.919	[1.117- 3.300]

PED, Psychiatric Emergency Department; MA, Methamphetamine. *p < 0.05; bold values also indicate statistical significance.

## 4 Discussion

### 4.1 Sociodemographic and admission characteristics of MA users in PED

The present study provides a comprehensive overview of the clinical characteristics of methamphetamine (MA) users admitted to a psychiatric emergency department (PED) in a major mental health center in Türkiye. Our findings highlight the high prevalence of acute psychotic symptoms, a wide spectrum of hallucinations and delusions, significant psychiatric comorbidity, and frequent use of three or more drugs among this population.

A significant majority of MA-related PED visits involved young males (84.2%), which is consistent with previous research (23, 31, 32). The predominance of male patients in our sample is consistent with international data indicating a higher prevalence of MA use among men (33, 34). In Türkiye, sociocultural barriers and stigma further limit women’s access to treatment, contributing to their underrepresentation in clinical samples (35). In our cohort, a large proportion of MA-related PED visits involved younger individuals, supporting earlier findings in the literature (20, 23, 31, 32, 36, 37). In our sample, a considerable proportion of MA-related PED presentations arrived via ambulance. Prior studies have similarly reported higher rates of ambulance use among substance-related psychiatric emergencies compared to other patient groups (38, 39).

These findings suggest that MA use continues to pose a significant challenge for PEDs particularly among young male individuals and highlight the importance of targeted prevention and intervention strategies for this demographic.

### 4.2 Clinical characteristics of MA users in PED

The clinical presentation of MA users admitted to the PED was characterized by a high prevalence of acute psychiatric symptoms. About two-thirds (65.5%) presented with psychotic symptoms, most commonly hallucinations and various delusion types. This finding is consistent with prior reports on the high frequency of psychosis in MA users (2, 40). The diversity and complexity of hallucinations and delusional content observed in this cohort reflect the heterogeneity of psychotic manifestations associated with MA intoxication, as described in the literature (41, 42). Dissociative symptoms, including depersonalization and derealization, were also commonly reported, further supporting the notion that MA use can profoundly disrupt perceptual and self-referential processes (43).

In addition to psychosis, rates of suicidal ideation (9.9%), attempts (5%), and self-harm (14.4%) were comparable to Turkish and international reports (24, 32, 44, 45). Insomnia (60.8%) and agitation (65.7%) further underline MA’s broad neuropsychiatric impact and the need for careful clinical management.

Use of three or more drugs was another notable feature in this population, with approximately 10% of patients using three or more psychoactive substances concurrently. Alcohol, cannabis, synthetic cannabinoids, opioids, and cocaine were among the substances most frequently used alongside MA, a pattern that is consistent with previous research (1, 20, 21, 46). The combined and potentially synergistic effects of these substances are likely to exacerbate neurotoxicity and intensify psychiatric symptoms, thereby complicating clinical management and outcomes (47, 48). For instance, the use of MA in individuals with opioid use disorder has the potential to exacerbate the adverse medical and social effects of psychoactive substance use. Moreover, it has been demonstrated that this practice can impede treatment outcomes, elevate rates of psychiatric and medical complications, and heighten the risks of infectious diseases and other adverse outcomes (36).

The presence of comorbid psychiatric disorders, including schizophrenia spectrum and psychotic disorders not otherwise specified, bipolar disorder, depression, anxiety, and substance-related psychosis, further complicates the clinical picture (1, 7, 23, 42, 47, 49, 50). In this sample, nearly 56% had a comorbid psychiatric disorder, including 26.1% with a psychotic spectrum disorder and 17.2% with a mood disorder. This high comorbidity in MA users has significant treatment implications, as co-occurring psychotic disorders and mood disorders are known to worsen clinical outcomes and increase the risk of emergency psychiatric admissions and hospitalization (16, 23). Beyond Türkiye, similar trends have been documented in other non-Western settings. A large-scale study from China reported that 57.6% of MA users presented with at least one psychiatric symptom, including psychosis, depression, and anxiety, while 8.3% exhibited all three simultaneously, underscoring the substantial burden of comorbidity in this population (13). Likewise, reports from Iran also emphasize the rising burden of MA-related psychosis as a major public health issue, suggesting that the psychiatric consequences of MA use are a widespread challenge beyond Western contexts (7).

In terms of acute management, parenteral APs (63.1%) and benzodiazepines (21%) were commonly administered, reflecting the severity of psychiatric disturbances in this population. Physical restraint was needed in about one-fourth of cases, reflecting the level of agitation and potential for violence, and underscoring the need for both pharmacological and non-pharmacological interventions in the acute management of MA-induced psychiatric symptoms, as well as the importance of staff training in de-escalation and non-coercive management strategies (23).

The data reveal a notable shift in clinical outcomes over the course of one year. While 33.9% of patients required hospitalization at their initial emergency department visit, a similar proportion—33.3% of those with available follow-up—were hospitalized within a year. This persistence in hospitalization rates suggests that, for many individuals, the challenges associated with methamphetamine use do not resolve after the first crisis but instead tend to recur. At the same time, there is a clear increase in the proportion of patients diagnosed with psychotic disorders. At admission, 9.0% of patients were diagnosed with substance-induced psychosis and 15.4% with other psychotic disorders, totaling 24.4%. By the end of the follow-up period, these rates had risen to 24.3% and 25.4%, respectively, among those with available data—meaning nearly half of the patients (49.7%) had a diagnosis of either substance-induced or other psychotic disorder after one year. This marked rise indicates that MA exposure is associated with a significant risk of developing persistent or new-onset psychotic symptoms over time. The proportion of patients without a psychiatric diagnosis at follow-up (24.6%) suggests that some may experience stabilization or remission, yet the majority continue to struggle with ongoing symptoms or new diagnoses. These patterns highlight the need for ongoing, individualized care and suggest that early intervention alone may not be sufficient to prevent future relapses or hospitalizations. Hence, implementation of routine mental health screening and referral protocols in PEDs could facilitate early intervention and reduce the risk of chronic psychiatric morbidity for. These findings highlight the importance of multidisciplinary approaches addressing both substance use and psychiatric comorbidities (1, 21, 47, 51). The variation in referral rates to the hospital’s ASUDDC further emphasizes the importance of individualized treatment pathways. Addressing both substance use and co-occurring psychiatric comorbidities in specialized, multidisciplinary settings appears critical for improving long-term outcomes in this population.

### 4.3 Predictors of suicidality and self-harm in MA users admitted to PED

A retrospective study of considerable scale, employing National Health Insurance Research Datasets (NHIRD) in Taiwan, revealed that MA users exhibited an elevated level of risk with respect to psychiatric comorbidities, such as depressive disorders, sleep disorders, medication-induced mental disorders, schizophrenia, and bipolar disorder (51). Accordingly, the prevalence ratio of hospital admissions involving both suicidal ideation and MA use is reported to be increased substantially, rising 16-fold between 2008 and 2019 (52). Suicide attempts are far more common among MA users, contributing to 25–50% of deaths in this group (53). The present study identified comorbid depression and self-harm as the strongest predictors for suicidal ideation and attempts. Furthermore, the presence of psychotic symptoms and comorbid psychiatric disorders were also associated with suicidality, consistent with findings reported in the broader literature (28, 42, 52, 54–56). These findings are not limited to Western populations. A study from Iraq demonstrated that crystal methamphetamine users face significantly elevated risks of suicidal ideation, particularly when presenting with visual hallucinations, episodes of aggression, or concurrent use of other illicit substances (54). Data from Saudi Arabia consistently indicate that MA users exhibit particularly high levels of suicidal behavior, with methamphetamine use identified as a strong independent predictor of suicidality (10, 14).

Psychiatric comorbidity was confirmed as a significant predictor of suicide attempts in our sample with comorbid mood and psychotic disorders being especially prominent. This is consistent with recent studies showing that MA users who die by suicide have higher rates of psychiatric comorbidities, particularly depression, psychosis, and bipolar disorder (19, 25, 57). Given the high prevalence of these comorbidities in our sample from Türkiye, structured psychiatric assessments are essential for early identification and intervention.

In our cohort, self-harm was the strongest predictor of suicide attempts (OR = 26.5), serving as a direct marker of imminent suicide risk. This finding is consistent with broader evidence showing that prior self-harm is one of the most robust predictors of future suicidal behavior across populations (56). Factors such as insomnia, agitation, and the presence of psychotic symptoms, along with concurrent polysubstance use, were also strongly associated with self-harm in our sample. Supporting this finding, a previous study from Türkiye reported significantly higher levels of self-harm among MA users with psychotic symptoms compared to those without (32). These observations highlight the need for comprehensive evaluation and integrated care addressing depression, psychosis, sleep problems, agitation, and polysubstance use in order to reduce suicidality risks.

Although depression, psychotic symptoms, insomnia, agitation, and self-harm are well-known predictors of suicidality in general psychiatric populations (58, 59), recent studies indicate that these factors are especially pronounced among individuals with MA use disorder (47, 51). In MA users, such symptoms often present more acutely and with greater severity, particularly during intoxication or withdrawal, and may lead to sudden suicidal ideation or self-harm (60, 61). The neurobiological effects of MA, including its impact on mood regulation and impulse control, appear to amplify these risks beyond what is typically seen in other groups (47, 60). Additionally, frequent use of three or more drugs use and fluctuating mental states complicate risk assessment and intervention (15, 51). While these predictors are not unique to MA users, their intensity and the clinical challenges they create in emergency settings highlight the need for tailored assessment and management strategies.

### 4.4 Predictors of aggression and physical restraint in MA users admitted to PED

The findings, supported by multivariate logistic regression analyses (see **Tables 6** and 7), reveal that the presence of psychotic symptoms, agitation, and self-harm are the most robust predictors for both aggressive behavior and the need for physical restraint during acute presentations. Consistent with international literature, psychotic symptoms significantly increased the odds of aggression (OR = 2.65) and physical restraint (OR = 6.10). Agitation was also a strong independent predictor for both outcomes (aggression: OR = 2.24; restraint: OR = 3.21, respectively). Self-harm, often under-recognized as a marker of acute behavioral dysregulation, was associated with a more than threefold increase in the risk of aggression (OR = 3.54) and a more than fourfold increase in the risk of physical restraint (OR = 4.47). These findings are in line with previous studies indicating that MA-induced psychosis and agitation are key drivers of violence and coercive interventions in PEDs (44, 47, 61–64). Regional data, including reports from Saudi Arabia and Iran, similarly highlight severe aggression among MA users, ranging from impulsivity to violent behavior (10, 65). Accordingly, the higher rates of comorbid psychiatric disorders and use of three or more drugs use observed in this cohort should not be underestimated in this context. Although these factors did not consistently emerge as statistically significant predictors in the multivariate models, their presence likely contributes to an elevated overall risk environment and should be carefully considered during clinical risk assessment. The heterogeneity of psychotic and dissociative symptoms further complicates risk management, underscoring the need for individualized interventions. The link between self-harm and outward aggression reflects the complex interplay of internalizing and externalizing violence seen in MA users (66). However, the high rates of physical restraint observed in the present study and others in this field raise ethical and practical concerns. There is an ongoing need for staff training in non-coercive management strategies, as well as the development of less restrictive alternatives.

### 4.5 Predictors of frequent PED visits, psychiatric hospitalization in a closed ward within one year of PED admission, and substance-induced psychosis in MA users admitted to PED

Consistent with international evidence, comorbid psychiatric disorders and a history of previous psychiatric hospitalization were strong predictors of frequent PED visits (67, 68). This mirrors evidence that diagnostic complexity and prior service use drive recurrent emergency department visits. These patterns are also reflected in Taiwanese data, where national studies showed high medical utilization among MA users (19), and emergency department studies further highlighted gender differences in drug-related presentations, including those associated with MA use (37). Unlike some North American and European cohorts, factors such as homelessness, polysubstance use, and female sex did not emerge as independent predictors in our analyses. It should be noted, however, that homelessness could not be assessed in our dataset as housing status was not routinely documented in our emergency department records and the low proportion of female patients may have limited the ability to detect sex-related effect. This divergence may be attributable to both methodological and contextual factors. In Türkiye, homelessness is relatively rare compared to Western countries, largely due to strong extended family structures, cultural expectations regarding familial support, and social safety nets that reduce the risk of individuals becoming unsheltered. As a result, emergency departments do not routinely document housing status, and the low prevalence of homelessness in both the general and clinical populations may have limited the ability to detect its association with frequent PED visits. Additionally, the underrepresentation of women in treatment-seeking populations may further contribute to these differences (69).

In the present study, older age and prior closed-ward admission are found to be predictors of psychiatric hospitalization within one year. The latter finding aligns with global evidence that inpatient “career” trajectories perpetuate further admissions (70–73). However, while international data often highlight younger age as a risk factor for hospitalization (73), the present findings indicate that, in Türkiye, older age was more strongly related to psychiatric hospitalization. This discrepancy may be explained by the unique dynamics of the Turkish healthcare and social context. Although the Turkish Civil Code grants physicians the authority to make involuntary admission decisions, in practice, clinicians frequently take family preferences into account. Studies have shown that Turkish families are closely involved in the decision-making process regarding psychiatric hospitalization, and concerns about stigma and the perceived burden of mental illness often influence these decisions (74–76). While direct data on age-specific attitudes are limited, clinical experience and some qualitative reports suggest families may be especially reluctant to hospitalize younger patients, due to concerns about stigma and future social consequences. This tendency may contribute to the lower hospitalization rates observed among younger individuals in our setting. These findings underscore the importance of considering local cultural norms and health system practices when interpreting age-related risk factors for psychiatric hospitalization.

In the cohort of the study, a subsequent diagnosis of substance-induced psychosis after one year was independently associated with male sex, the presence of psychotic symptoms at initial presentation, agitation, aggression, and a history of closed-ward psychiatric admission. However, the magnitude of the male excess observed in the sample and the emergence of agitation and aggression as longitudinal predictors diverge from most international studies. Baseline psychotic symptoms and prior hospitalization consistently predict later psychosis in MA users, underscoring the role of early morbidity and acute symptom severity (60, 77). Reports from Iran caution that psychotic symptoms in MA users may persist in a subset of patients, making the distinction between transient and persistent forms of methamphetamine-associated psychosis (MAP) increasingly important (7). Consistent with these findings, our results also suggest that MA-related psychiatric sequelae often extend beyond short-lived episodes, with a substantial risk of chronicity, recurrence or progression, in line with previous research on methamphetamine-associated psychosis (1, 8, 12). Prior research including evidence from non-Western countries such as China and Taiwan, more commonly identifies early-onset MA use, cannabis co-use, and family history of psychosis as primary risk factors for substance induced psychosis (78–81). Longitudinal data from Taiwan also demonstrate the severe trajectory of MAP. In one cohort, nearly 40% of patients required rehospitalization during follow-up, over one-third received a schizophrenia diagnosis due to persistent psychosis, and more than half experienced relapse of psychotic symptoms, underscoring the substantial risk of chronic or recurrent MAP (82). Male predominance in our cohort may have inflated risk estimates, a limitation also reported in other regional samples (69, 83).

### 4.6 Implications for psychiatric emergency policy and long-term care pathways

Our study stands out as one of the first to show, through one-year follow-up data, that psychotic disorders and hospital admissions among MA users not only persist but may even increase after the initial emergency department visit. This pattern highlights a pressing need for more than just acute crisis management; it calls for the creation of structured, long-term follow-up and care pathways within PEDs—an area that has received little attention in previous research. In light of these findings, the authors suggest that health policymakers and administrators focus on early identification, rapid triage, and the development of integrated care models tailored to the unique needs of this vulnerable group. It will also be crucial to invest in staff training for de-escalation, foster multidisciplinary teamwork, and ensure that mental health and addiction services are closely linked within emergency settings. By putting these evidence-based recommendations into practice PEDs can improve patient outcomes, make better use of resources, and play a more effective role in addressing the growing public health challenge posed by MA use.

### 4.7 Strengths and limitations

To our knowledge, this study represents one of the most comprehensive examinations of methamphetamine users admitted to a psychiatric emergency department in Türkiye—a nation uniquely positioned between Europe and Asia. Drawing on a large and diverse patient population from a leading mental health center, our findings are both relevant and broadly applicable. The systematic extraction of data from electronic health records enabled a thorough assessment of sociodemographic, clinical, and treatment-related characteristics. Notably, the inclusion of a one-year follow-up period allowed us to evaluate both acute presentations and longer-term outcomes, such as hospitalization and repeated emergency visits. Employing multivariate analyses to identify independent predictors of adverse outcomes further strengthened the study’s methodological rigor. By reflecting the real-world complexity of clinical practice—including high rates of comorbidity and frequent use of multiple substances—this research addresses a significant gap in the regional literature and offers evidence to inform both clinical care and health policy in psychiatric emergency settings.

Nevertheless, several limitations should be considered. The retrospective design may have resulted in underreporting of certain symptoms, and the absence of structured diagnostic interviews introduces the possibility of missing or inaccurately recorded information in the electronic health records. Due to the acute nature of emergency department admissions, detailed information on the duration and cumulative pattern of MA use was not consistently available, limiting our ability to analyze its impact on clinical outcomes. As a single-center study, the findings may not be fully generalizable to other settings with different healthcare systems or patient populations. Furthermore, because the sample was drawn exclusively from a tertiary psychiatric emergency department in Istanbul, it may be biased toward individuals with more acute and disruptive presentations. Accordingly, the findings cannot be generalized to all MA users in Türkiye. Nevertheless, ERSHEAH serves a metropolitan catchment area of over 6 million people and is the only public psychiatric hospital with a PED on the Asian side of Istanbul, accepting referrals from surrounding provinces. This provides an important, though still regionally limited, picture of MA users requiring psychiatric emergency care. The generalizability of our findings is limited by the demographic composition of the sample, which was predominantly male (84.2%). Consequently, the conclusions may not be applicable to women, adolescents, or older adults. The relatively high prevalence of polysubstance use in our sample also complicates efforts to isolate the specific effects of methamphetamine. Diagnoses were based on ICD-10 codes assigned during routine clinical care, rather than structured research interviews, which may affect diagnostic reliability. Additionally, the cross-sectional nature of the admission data limits our ability to draw causal inferences between methamphetamine use and the psychiatric outcomes observed, and longitudinal research is needed to better understand symptom progression and the effectiveness of various treatment approaches over time. Given the number of statistical tests performed, the possibility of Type I error cannot be excluded. Finally, although 2022 was selected as a representative year, it followed the peak of the COVID-19 pandemic, which may have influenced mental health trends and patterns of service utilization.

## 5 Conclusions

Patients admitted to PE in Türkiye—a country bridging Europe and Asia—frequently present with severe acute psychiatric symptoms, often in the context of polysubstance use and significant psychiatric comorbidities. Routine screening for depression and self-harm in methamphetamine users at PEDs may facilitate earlier intervention and improved clinical outcomes. The high rates of recurrent emergency visits and hospitalizations observed in this population underscore the chronic and relapsing nature of MA-related psychiatric disturbances. The prevalence of psychiatric comorbidities and repeated emergency presentations indicates an urgent need for integrated clinical pathways and targeted interventions within emergency settings, as psychiatric comorbidity has been identified as a key predictor of adverse outcomes. These findings highlight the necessity for care models that address both acute symptoms and long-term management. Long-term care for MA users should encompass not only substance use treatment but also ongoing management of psychiatric comorbidities, as both are critical for optimizing patient outcomes. Further research is warranted to evaluate interventions that may enhance outcomes and reduce the burden on PEDs for this high-risk group.

## Data Availability

The raw data supporting the conclusions of this article will be made available by the authors, without undue reservation.

## References

[1] McKetin R Degenhardt L Shanahan M Baker AL Lee NK Lubman DI. Health service utilisation attributable to methamphetamine use in Australia: Patterns, predictors and national impact. Drug Alcohol Rev. (2018) 37:196–204. doi: 10.1111/DAR.12518, PMID: 28294443

[2] Cruickshank CC Dyer KR. A review of the clinical pharmacology of methamphetamine. Addiction. (2009) 104:1085–99. doi: 10.1111/J.1360-0443.2009.02564.X, PMID: 19426289

[3] EMCDDA. Synthetic stimulants-The current situation in Europe (European Drug Report 2023) (2023). Available online at: https://www.euda.europa.eu/publications/european-drug-report/2023/synthetic-stimulants_en (Accessed September 12, 2025).

[4] Volkow ND. Collision of the COVID-19 and addiction epidemics. Ann Intern Med. (2020) 173:61–2. doi: 10.7326/M20-1212, PMID: 32240293 PMC7138334

[5] European Union Drug Agency. European Drug Report (2025). Available online at: https://www.euda.europa.eu/publications/european-drug-report/2025/synthetic-stimulants_en (Accessed July 1, 2025).

[6] Armoon B Fleury MJ Griffiths MD Bayani A Mohammadi R Ahounbar E. Emergency department use, hospitalization, and their sociodemographic determinants among patients with substance-related disorders: A worldwide systematic review and meta-analysis. Subst Use Misuse. (2023) 58:331–45. doi: 10.1080/10826084.2022.2161313, PMID: 36592043

[7] Alam Mehrjerdi Z Barr AM Noroozi A. Methamphetamine-associated psychosis: a new health challenge in Iran. DARU J Pharm Sci. (2013) 21:1–3. doi: 10.1186/2008-2231-20-73, PMID: 23577655 PMC3637332

[8] Harris D Batki SL. Stimulant psychosis: Symptom profile anti acute clinical course. Am J Addict. (2000) 9:28–37. doi: 10.1080/10550490050172209, PMID: 10914291

[9] Fulde GWO Forster SL. The impact of amphetamine-type stimulants on emergency services. Curr Opin Psychiatry. (2015) 28:275–9. doi: 10.1097/YCO.0000000000000171, PMID: 26001917

[10] Ali MI Rashad MM Alzain NM Al-Awad FA Alzaharani MA Alshamarani AS. Impulsiveness, suicide, and aggression in a sample of patients with disorders of methyl amphetamine use. J Family Community Med. (2024) 31:257–64. doi: 10.4103/JFCM.JFCM_4_24, PMID: 39176013 PMC11338392

[11] Wearne TA Cornish JL. A comparison of methamphetamine-induced psychosis and schizophrenia: A review of positive, negative, and cognitive symptomatology. Front Psychiatry. (2018) 9:491. doi: 10.3389/fpsyt.2018.00491, PMID: 30364176 PMC6191498

[12] Chen CK Lin SK Sham PC Ball D Loh EW Hsiao CC. Pre-morbid characteristics and co-morbidity of methamphetamine users with and without psychosis. Psychol Med. (2003) 33:1407–14. doi: 10.1017/S0033291703008353, PMID: 14672249

[13] Ma J Sun XJ Wang RJ Wang TY Su MF Liu MX. Profile of psychiatric symptoms in methamphetamine users in China: Greater risk of psychiatric symptoms with a longer duration of use. Psychiatry Res. (2018) 262:184–92. doi: 10.1016/j.psychres.2018.02.017, PMID: 29453037

[14] Aljadani AH Abouzed M Adam H Alrasheedy BB Alarfaj M Alshammari SR. Methamphetamine use and suicide risk: a comprehensive case–control study. Front Public Health. (2025) 13:1593935. doi: 10.3389/fpubh.2025.1593935, PMID: 40777657 PMC12328327

[15] Zito MF Fei Z Zhu Y Clingan SE Marder SR Mooney LJ. Psychosis among individuals with methamphetamine use disorder is associated with elevated rates of hospitalizations and emergency department visits across an academic health care system. J Subst Use Addict Treat. (2023) 151:209033. doi: 10.1016/J.JOSAT.2023.209033, PMID: 37011880

[16] Unadkat A Subasinghe S Harvey RJ Castle DJ. Methamphetamine use in patients presenting to emergency departments and psychiatric inpatient facilities: what are the service implications? Australas Psychiatry. (2018) 27:14–7. doi: 10.1177/1039856218810155, PMID: 30382752

[17] Usher K Jackson D Woods C Sayers J Kornhaber R Cleary M. Safety, risk, and aggression: Health professionals’ experiences of caring for people affected by methamphetamine when presenting for emergency care. Int J Ment Health Nurs. (2017) 26:437–44. doi: 10.1111/INM.12345, PMID: 28960736

[18] Richards JR Hawkins JA Acevedo EW Laurin EG. The care of patients using methamphetamine in the emergency department: perception of nurses, residents, and faculty. Subst Abus. (2019) 40:95–101. doi: 10.1080/08897077.2018.1449170, PMID: 29595368

[19] Lee WC Chang HM Huang MC Pan CH Su SS Tsai SY. Increased medical utilization and psychiatric comorbidity following a new diagnosis of methamphetamine use disorder. Am J Drug Alcohol Abuse. (2022) 48:245–54. doi: 10.1080/00952990.2021.1979990, PMID: 34670448

[20] Miller J Atala R Sarangarm D Tohen M Sharma S Bhatt S. Methamphetamine abuse trends in psychiatric emergency services: A retrospective analysis using big data. Community Ment Health J. (2020) 56:959–62. doi: 10.1007/S10597-020-00563-1/METRICS, PMID: 31997123

[21] Winkelman TNA Admon LK Jennings L Shippee ND Richardson CR Bart G. Evaluation of amphetamine-related hospitalizations and associated clinical outcomes and costs in the United States. JAMA Netw Open. (2018) 1:e183758. doi: 10.1001/JAMANETWORKOPEN.2018.3758, PMID: 30646256 PMC6324446

[22] Murphy CE IV Wang RC Coralic Z Lai AR Raven M. Association between methamphetamine use and psychiatric hospitalization, chemical restraint, and emergency department length of stay. Acad Emergency Med. (2020) 27:1116–25. doi: 10.1111/acem.14094, PMID: 32713087

[23] Pasic J Russo JE Ries RK Roy-Byrne PP. Methamphetamine users in the psychiatric emergency services: a case-control study. Am J Drug Alcohol Abuse. (2007) 33:675–86. doi: 10.1080/00952990701522732, PMID: 17891660

[24] Bayazit H Baronia R Wakefield SM. Methamphetamine intoxication and suicidal ideation/behavior in the emergency department. Curr Med Res Opin. (2024) 40:849–54. doi: 10.1080/03007995.2024.2333429, PMID: 38511972

[25] Salo R Flower K Kielstein A Leamon MH Nordahl TE Galloway GP. Psychiatric comorbidity in methamphetamine dependence. Psychiatry Res. (2011) 186:356–61. doi: 10.1016/J.PSYCHRES.2010.09.014, PMID: 21055832 PMC3058719

[26] Shearer RD Howell BA Bart G Winkelman TNA. Substance use patterns and health profiles among US adults who use opioids, methamphetamine, or both, 2015-2018. Drug Alcohol Depend. (2020) 214:108162. doi: 10.1016/J.DRUGALCDEP.2020.108162, PMID: 32652380 PMC8147519

[27] Wang L Min JE Krebs E Evans E Huang D Liu L. Polydrug use and its association with drug treatment outcomes among primary heroin, methamphetamine, and cocaine users. Int J Drug Policy. (2017) 49:32–40. doi: 10.1016/J.DRUGPO.2017.07.009, PMID: 28888099 PMC5681890

[28] Marshall BDL Werb D. Health outcomes associated with methamphetamine use among young people: A systematic review. Addiction. (2010) 105:991–1002. doi: 10.1111/J.1360-0443.2010.02932.X, PMID: 20659059

[29] McKenna B McEvedy S Kelly K Long B Anderson J Dalzell E. Association of methamphetamine use and restrictive interventions in an acute adult inpatient mental health unit: A retrospective cohort study. Int J Ment Health Nurs. (2017) 26:49–55. doi: 10.1111/INM.12283, PMID: 27860236

[30] Mutlu E Alaei A Tracy M Waye K Cetin MK Alaei K. Correlates of injection drug use among individuals admitted to public and private drug treatment facilities in Turkey. Drug Alcohol Depend. (2016) 164:71–81. doi: 10.1016/J.DRUGALCDEP.2016.04.032, PMID: 27173661

[31] Nakamoto M Onoye J Kiyokawa M Takeshita J Lu B. Methamphetamine use in psychiatric emergency services and among asian american and pacific islander populations. J Addict Med. (2024) 18:657–62. doi: 10.1097/ADM.0000000000001335, PMID: 38869174

[32] ataliyev İ Coskun MN Topcuoğlu M Erdoğan A Kulaksızoğlu B. Sociodemographic and clinical characteristics of patients admitted to an addiction center with methamphetamine use. J Subst Use. (2024) 1–5. doi: 10.1080/14659891.2024.2446914

[33] Jones CM Compton WM Mustaquim D. Morbidity and mortality weekly report patterns and characteristics of methamphetamine use among adults-United States, 2015-2018. MMWR Morb Mortal Wkly Rep. (2020) 69:317–23. doi: 10.15585/mmwr.mm6912a1, PMID: 32214077 PMC7725509

[34] EMCDDA. European Drug Report 2023. Luxembourg: Publications Office of the European Union (2023).

[35] Eslek AY Ozsahin N Eslek I Ayer A. Drawing attention to addiction in women by evaluating female inpatients hospitalized in MRSHH-AMATEM. Dusunen Adam - J Psychiatry Neurological Sci. (2015) 28:179–80. doi: 10.5350/DAJPN2015280213

[36] Chawarski MC Hawk K Edelman EJ O’Connor P Owens P Martel S. Use of amphetamine-type stimulants among emergency department patients with untreated opioid use disorder. Ann Emerg Med. (2020) 76:782–7. doi: 10.1016/J.ANNEMERGMED.2020.06.046, PMID: 32782084 PMC8048036

[37] Weng TI Chen LY Chen HY Yu JH Su YJ Liu SW. Gender differences in clinical characteristics of emergency department patients involving illicit drugs use with analytical confirmation. J Formosan Med Assoc. (2022) 121:1832–40. doi: 10.1016/J.JFMA.2022.03.007, PMID: 35365378

[38] Bunting PJ Fulde GWO Forster SL. Comparison of crystalline methamphetamine (“ice”) users and other patients with toxicology-related problems presenting to a hospital emergency department. Med J Aust. (2007) 187:564–6. doi: 10.5694/J.1326-5377.2007.TB01417.X, PMID: 18021044

[39] Gray SD Fatovich DM McCoubrie DL Daly FF. Amphetamine-related presentations to an inner-city tertiary emergency department: a prospective evaluation. Med J Aust. (2007) 186:336–9. doi: 10.5694/J.1326-5377.2007.TB00932.X, PMID: 17407428

[40] Sekine Y Iyo M Ouchi Y Matsunaga T Tsukada H Okada H. Methamphetamine-related psychiatric symptoms and reduced brain dopamine transporters studied with PET. Am J Psychiatry. (2001) 158:1206–14. doi: 10.1176/APPI.AJP.158.8.1206/ASSET/IMAGES/J57F6.JPEG, PMID: 11481152

[41] McKetin R McLaren J Lubman DI Hides L. The prevalence of psychotic symptoms among methamphetamine users. Addiction. (2006) 101:1473–8. doi: 10.1111/J.1360-0443.2006.01496.X, PMID: 16968349

[42] Zweben JE Cohen JB Christian D Galloway GP Salinardi M Parent D. Psychiatric symptoms in methamphetamine users. Am J Addict. (2004) 13:181–90. doi: 10.1080/10550490490436055, PMID: 15204668

[43] Sekine Y Minabe Y Ouchi Y Takei N Iyo M Nakamura K. Association of dopamine transporter loss in the orbitofrontal and dorsolateral prefrontal cortices with methamphetamine-related psychiatric symptoms. Am J Psychiatry. (2003) 160:1699–701. doi: 10.1176/APPI.AJP.160.9.1699/ASSET/IMAGES/L025F1.JPEG, PMID: 12944350

[44] Jones R Woods C Barker R Usher K. Patterns and features of methamphetamine-related presentations to emergency departments in QLD from 2005 to 2017. Int J Ment Health Nurs. (2019) 28:833–44. doi: 10.1111/INM.12618, PMID: 31179592

[45] Cloutier RL Hendrickson RG Fu R Blake B. Methamphetamine-related psychiatric visits to an urban academic emergency department: an observational study. J Emerg Med. (2013) 45:136–42. doi: 10.1016/J.JEMERMED.2012.11.094, PMID: 23561310

[46] Alniak I Ulusoy S Cebeci BA. The use of methamphetamine in the probation population. Dusunen Adam: J Psychiatry Neurological Sci. (2022) 35:2–12. doi: 10.14744/DAJPNS.2022.00166

[47] McKetin R Leung J Stockings E Huo Y Foulds J Lappin JM. Mental health outcomes associated with of the use of amphetamines: A systematic review and meta-analysis. EClinicalMedicine. (2019) 16:81–97. doi: 10.1016/J.ECLINM.2019.09.014/ATTACHMENT/121D9CC8-CC9A-4541-A66D-7BAD082FE85D/MMC1.DOCX 31832623 PMC6890973

[48] Edinoff AN Kaufman SE Green KM Provenzano DA Lawson J Cornett EM. Methamphetamine use: A narrative review of adverse effects and related toxicities. Health Psychol Res. (2022) 10. doi: 10.52965/001c.38161, PMID: 36118981 PMC9476235

[49] Jones R Woods C Usher K. Rates and features of methamphetamine-related presentations to emergency departments: An integrative literature review. J Clin Nurs. (2018) 27:2569–82. doi: 10.1111/JOCN.14493, PMID: 29679414

[50] Lineberry TW Bostwick JM. Methamphetamine abuse: A perfect storm of complications. Mayo Clin Proc. (2006) 81:77–84. doi: 10.4065/81.1.77, PMID: 16438482

[51] Lee WC Chang HM Huang MC Pan CH Su SS Tsai SY. Healthcare utilization and psychiatric and physical comorbidities before suicide mortality in patients with methamphetamine use disorder: A nationwide case–control study. Addictive Behav. (2022) 126. doi: 10.1016/j.addbeh.2021.107192, PMID: 34839069

[52] Xing DG Horan T Bhuiyan MS Faisal ASM Densmore K Murnane KS. Social-geographic disparities in suicidal ideations among methamphetamine users in the USA. Psychiatry Res. (2023) 329. doi: 10.1016/j.psychres.2023.115524, PMID: 37852161 PMC10841467

[53] Darke S Kaye S Duflou J Lappin J. Completed suicide among methamphetamine users: A national study. Suicide Life Threat Behav. (2019) 49:328–37. doi: 10.1111/SLTB.12442, PMID: 29444345

[54] Al-Imam A Motyka MA Hoffmann B Al-Ka’aby H Younus M Al-Hemiary N. Risk factors of suicidal ideation in Iraqi crystal methamphetamine users. Brain Sci. (2023) 13. doi: 10.3390/BRAINSCI13091279, PMID: 37759880 PMC10526952

[55] Chiang M Lombardi D Du J Makrum U Sitthichai R Harrington A. Methamphetamine-associated psychosis: Clinical presentation, biological basis, and treatment options. Hum Psychopharmacol. (2019) 34. doi: 10.1002/HUP.2710, PMID: 31441135

[56] Simon GE Johnson E Lawrence JM Rossom RC Ahmedani B Lynch FL. Predicting suicide attempts and suicide deaths following outpatient visits using electronic health records. Am J Psychiatry. (2018) 175:951–60. doi: 10.1176/APPI.AJP.2018.17101167, PMID: 29792051 PMC6167136

[57] Espiridion ED Charron L. Methamphetamine use and suicide: A case report and brief review of literature. Cureus. (2024) 16. doi: 10.7759/CUREUS.64835, PMID: 39156401 PMC11330268

[58] Fazel S Runeson B. Suicide. N Engl J Med. (2020) 382:266. doi: 10.1056/NEJMRA1902944, PMID: 31940700 PMC7116087

[59] Hawton K van Heeringen K. Suicide. Lancet. (2009) 373:1372–81. doi: 10.1016/S0140-6736(09)60372-X, PMID: 19376453

[60] Arunogiri S Foulds JA McKetin R Lubman DI. A systematic review of risk factors for methamphetamine-associated psychosis. Aust New Z J Psychiatry. (2018) 52:514–29. doi: 10.1177/0004867417748750/SUPPL_FILE/APPENDIX_2.DOCX, PMID: 29338289

[61] Glasner-Edwards S Mooney LJ. Methamphetamine psychosis: Epidemiology and management. CNS Drugs. (2014) 28:1115–26. doi: 10.1007/S40263-014-0209-8, PMID: 25373627 PMC5027896

[62] Mcketin R Lubman DI Najman JM Dawe S Butterworth P Baker AL. Does methamphetamine use increase violent behaviour? Evidence from a prospective longitudinal study. Addiction. (2014) 109:798–806. doi: 10.1111/ADD.12474, PMID: 24400972

[63] McKetin R Lubman DI Baker AL Dawe S Ali RL. Dose-related psychotic symptoms in chronic methamphetamine users: evidence from a prospective longitudinal study. JAMA Psychiatry. (2013) 70:319–24. doi: 10.1001/JAMAPSYCHIATRY.2013.283, PMID: 23303471

[64] Zhang L Sun Y Wang J Zhang M Wang Q Xie B. Dopaminergic dominance in the ventral medial hypothalamus: A pivotal regulator for methamphetamine-induced pathological aggression. Prog Neuropsychopharmacol Biol Psychiatry. (2024) 132:110971. doi: 10.1016/J.PNPBP.2024.110971, PMID: 38365104

[65] Zarghami M. Methamphetamine has changed the profile of patients utilizing psychiatric emergency services in Iran. Iran J Psychiatry Behav Sci. (IJPBS) (2011) 5:1–5.

[66] Darke S Kaye S McKetin R Duflou J. Major physical and psychological harms of methamphetamine use. Drug Alcohol Rev. (2008) 27:253–62. doi: 10.1080/09595230801923702, PMID: 18368606

[67] Richards JR Placone TW Wang CG van der Linden MC Derlet RW Laurin EG. Methamphetamine, amphetamine, and MDMA use and emergency department recidivism. J Emerg Med. (2020) 59:320–8. doi: 10.1016/J.JEMERMED.2020.04.051, PMID: 32546441

[68] Bahji A Altomare J Sapru A Haze S Prasad S Egan R. Predictors of hospital admission for patients presenting with psychiatric emergencies: A retrospective, cohort study. Psychiatry Res. (2020) 290:113149. doi: 10.1016/J.PSYCHRES.2020.113149, PMID: 32512355

[69] Franke AG Neumann S Proebstl L Kamp F Hager L Manz K. Psychiatric comorbidity and psychopathology of methamphetamine users—Are there gender differences? Int J Ment Health Addict. (2023) 21:2632–49. doi: 10.1007/S11469-021-00743-4/FIGURES/2

[70] Morel D Yu KC Liu-Ferrara A Caceres-Suriel AJ Kurtz SG Tabak YP. Predicting hospital readmission in patients with mental or substance use disorders: A machine learning approach. Int J Med Inform. (2020) 139:104136. doi: 10.1016/J.IJMEDINF.2020.104136, PMID: 32353752

[71] Olsson MO Öjehagen A Brådvik L Håkansson A. Predictors of psychiatric hospitalization in ex-prisoners with substance use problems: A data-linkage study. J Drug Issues. (2015) 45:202–13. doi: 10.1177/0022042615575374

[72] Turkington A Stirling E. Predictors of readmission to an acute psychiatric inpatient unit. BJPsych Open. (2024) 10:S208–9. doi: 10.1192/BJO.2024.518

[73] Moos RH Mertens JR Brennan PL. Rates and predictors of four-year readmission among late-middle-aged and older substance abuse patients. J Stud Alcohol. (1994) 55:561–70. doi: 10.15288/JSA.1994.55.561, PMID: 7990466

[74] Dikeç G Uzunoğlu G Gümüş F. Stigmatization experiences of Turkish parents of patients hospitalized in child and adolescent psychiatric clinics. Perspect Psychiatr Care. (2019) 55:336–43. doi: 10.1111/PPC.12361, PMID: 30680723

[75] Eker D. Attitudes toward mental illness: Recognition, desired social distance, expected burden and negative influence on mental health among Turkish freshmen. Soc Psychiatry Psychiatr Epidemiol. (1989) 24:146–50. doi: 10.1007/BF01788024, PMID: 2500713

[76] Arkar H Eker D. Influence of having a hospitalized mentally ill member in the family on attitudes toward mental patients in Turkey. Soc Psychiatry Psychiatr Epidemiol. (1992) 27:151–5. doi: 10.1007/BF00788762, PMID: 1621141

[77] McKetin R. Methamphetamine psychosis: insights from the past. Addiction. (2018) 113:1522–7. doi: 10.1111/ADD.14170, PMID: 29516555

[78] Usher K Clough A Woods C Robertson J. Is there an ice epidemic in Australia? Int J Ment Health Nurs. (2015) 24:283–5. doi: 10.1111/INM.12155, PMID: 26245520

[79] Kalayasiri R Verachai V Gelernter J Mutirangura A Malison RT. Clinical features of methamphetamine-induced paranoia and preliminary genetic association with DBH –1021C→T in a Thai treatment cohort. Addict (Abingdon England). (2014) 109:965. doi: 10.1111/ADD.12512, PMID: 24521142 PMC4018411

[80] Gan H Zhao Y Jiang H Zhu Y Chen T Tan H. A research of methamphetamine induced psychosis in 1,430 individuals with methamphetamine use disorder: Clinical features and possible risk factors. Front Psychiatry. (2018) 9:551/BIBTEX. doi: 10.3389/FPSYT.2018.00551/BIBTEX, PMID: 30459651 PMC6232294

[81] Lin CH Chen JJ Chan CH. Comparison of psychiatric and clinical profiles between people who use synthetic cathinones and methamphetamine: A matched case-control study. J Clin Psychopharmacol. (2023) 43:122–30. doi: 10.1097/JCP.0000000000001649, PMID: 36706307 PMC9988231

[82] Kittirattanapaiboon P Mahatnirunkul S Booncharoen H Thummawomg P Dumrongchai U Chutha W. Long-term outcomes in methamphetamine psychosis patients after first hospitalisation. Drug Alcohol Rev. (2010) 29:456–61. doi: 10.1111/j.1465-3362.2010.00196.x, PMID: 20636664

[83] Clifford B Van Gordon K Magee F Malone V Siefried KJ Graham D. There’s a big tag on my head”: exploring barriers to treatment seeking with women who use methamphetamine in Sydney, Australia. BMC Health Serv Res. (2023) 23:1–9. doi: 10.1186/S12913-023-09125-Z, PMID: 36793060 PMC9933255

